# Fish Geometry and Electric Organ Discharge Determine Functional Organization of the Electrosensory Epithelium

**DOI:** 10.1371/journal.pone.0027470

**Published:** 2011-11-11

**Authors:** Juan Ignacio Sanguinetti-Scheck, Eduardo Federico Pedraja, Esteban Cilleruelo, Adriana Migliaro, Pedro Aguilera, Angel Ariel Caputi, Ruben Budelli

**Affiliations:** 1 Departamento de Biología Celular y Molecular, Facultad de Ciencias, Universidad de la República, Montevideo, Uruguay; 2 Departamento de Neurociencias Integrativa, Instituto de Investigaciones Biológicas Clemente Estable, Montevideo, Uruguay; University of Maryland, United States of America

## Abstract

Active electroreception in *Gymnotus omarorum* is a sensory modality that perceives the changes that nearby objects cause in a self generated electric field. The field is emitted as repetitive stereotyped pulses that stimulate skin electroreceptors. Differently from mormyriformes electric fish, gymnotiformes have an electric organ distributed along a large portion of the body, which fires sequentially. As a consequence shape and amplitude of both, the electric field generated and the image of objects, change during the electric pulse. To study how *G. omarorum* constructs a perceptual representation, we developed a computational model that allows the determination of the self-generated field and the electric image. We verify and use the model as a tool to explore image formation in diverse experimental circumstances. We show how the electric images of objects change in shape as a function of time and position, relative to the fish's body. We propose a theoretical framework about the organization of the different perceptive tasks made by electroreception: 1) At the head region, where the electrosensory mosaic presents an electric fovea, the field polarizing nearby objects is coherent and collimated. This favors the high resolution sampling of images of small objects and perception of electric color. Besides, the high sensitivity of the fovea allows the detection and tracking of large faraway objects in rostral regions. 2) In the trunk and tail region a multiplicity of sources illuminate different regions of the object, allowing the characterization of the shape and position of a large object. In this region, electroreceptors are of a unique type and capacitive detection should be based in the pattern of the afferents response. 3) Far from the fish, active electroreception is not possible but the collimated field is suitable to be used for electrocommunication and detection of large objects at the sides and caudally.

## Introduction

Electroreceptive fish detect nearby objects by processing the information contained in the distribution of electric currents through their skin [1] . In weakly electric fish these currents are self-generated by the Electric Organ Discharge (EOD) with a species-specific waveform. The field generated is disturbed by surrounding objects modifying the basal transepidermal voltage or current density: this distortion (the difference or quotient between the transepidermal voltage or current density, through the fish skin, in the presence and the absence of the object) was defined as the electric image and allows the fish to infer parameters of the scene, as shape and distance [2], [3], [4]. Either transepidermal voltage or current densities have been considered to be the specific stimuli for the electroreceptors embedded in the fish skin [5]. These receptors transduce and encode the electric image into a neural image, which is the first neural representation of the external world [6].

In electrosensory perception, each object generates a signal that results from the deformation it induces on the fish's electric field. This deformation is a virtual field, called “object perturbing field” by Lissmann and Machin (1958) [1]. The object perturbing field is not directly measurable, but it is computable from the electric field in the presence of the object minus the electric field in its absence, called “basal field.” As any electric field, the object perturbing field can be considered as caused by a distribution of electric sources on the borders of the object: the “stamp” [7] (Caputi et al, 2008). This stamp is the consequence of the object's “imprimence” when it is in the presence of the electric field [1]. These sources generate a change in the field that interacts with other objects. Thus, the effect of a given object not only generates its own image but also modifies the images of other objects [8], [9].

Evolution has adopted different strategies for the generation of the EODs: continuous sine-wave-like or pulsatile. These two strategies are represented in the two different families of fishes evolved independently in Africa and America. Electric organs have different locations and anatomic plans: concentrated or distributed along the fish body [10]. The temporal and spatial dynamics of the EOD depend on the anatomy and position of the electric organ (EO) in the body, innervations pattern, the shape of the body, the electrical properties of the tissues surrounding the EO and the conductivity of the medium.

Concentrated organs (as those of *G. petersii*) produce fields and images with the same temporal shape everywhere. The amplitudes vary depending on the distance to the EO and to the fish's body [2]. In distributed organs as those of *G omarorum*, (a new described species previously indentified in most studies on EO as *G carapo*), sources located at different positions along the EO contribute with different temporal shapes and amplitudes reflecting the innervation patterns and sequence of activation of the electrogeneration units. Consequently, fish with distributed organs (as *G. omarorum*) produce fields and images with site specific temporal shapes. [11], [12], [13].

Theoretical analysis of image generation has yielded models that predict with acceptable accuracy the electrosensory stimuli. The first theoretical model focused on the effects that an object causes on the electric field [1]. The importance of the fish's body physical properties, geometry, resistance and the characteristics of the sources, were later taken into account [14], [15], [16]. These, essentially finite element models, allowed us to estimate the electric images of single resistive or capacitive objects [5]. These models are still been used and yield useful predictions and insights for electrolocation[17], [18]. Assad (1997) and Rasnow (1996) [19], [20] introduced the Boundary Element Methods (BEM) to predict the field and images of objects. Rother et al (2003) [5] merge realistic models based on physical measurements of the fish's body and sources with BEM to yield a precise prediction of electric image in *G. petersii*. This model allowed also evaluating the role of the fish's body and skin resistance in active electrolocation [6].

In fish with concentrated EO as *G. petersii* the field can be modeled as produced by a single dipole at the tail of the fish, with its amplitude varying as a function of time. As a consequence the field at any point has the same direction and the same temporal course.

Fish with distributed organs pose larger difficulties because of the complexity of the EO and the EOD. Since the discharge can change rapidly in time and space, modeling must contemplate these two variables. This is the focus of the present article, where we use a model of the field and electric images to understand image formation in *G. omarorum*. Our simulations indicate the existence of three electrosensory zones: a head zone in which the basal field is collimated (*i.e.,* almost in a fixed direction along the EOD) and coherent (i.e. it has the same time course); a trunk and tail zone in which the field is neither collimated nor coherent and a far field zone where the field is collimated but is not large enough to generate images of objects placed in such zone.

As happens in *G petersii*, the shape of the head's local EOD (LEOD) produced in the presence of a resistive object will change in amplitude but not in shape and the image is defined by a function: the change in amplitude of the LEOD in the presence and the absence of the object mapped on the skin surface. When the object is capacitive, the LEOD changes in amplitude and in shape. Thus, the images inform, not only of intensity, but also about some qualia, we called “electric color” [6], [21]. In addition the collimation improves the efficiency of the high resolution mosaic present at the perioral region.

At the trunk and tail zone the multiple sources allow for the “illumination” of large objects from different directions. The resulting image is a more faithful representation than the one achieved by illuminating the object from a localized source.

Finally, the far electric fields may carry information to conspecifics or can be modified by very large contextual elements like water surface.

These differential characteristics integrate a theoretical scheme about how electrosensibility is used by gymnotiform fish to process information from the environment.

## Methods

### The model

Electric images and fields were calculated using the BEM's based software that has been previously described [5], [6].

We defined the scene by setting the geometry and location of the electric fish and the objects with their different impedances, shapes and sizes. Water, internal, and skin conductivities were specified according to experimental measurements [15]. Objects shapes, including the fish body, were approximated by a surface composed by triangles. Although the fish shape is kept constant throughout this article, the model allows its modification if required. For each scene, electric fields and images were calculated. Graphics were made by MATLAB (MathWorks, Natick, MA, USA) standard subroutines.

The geometry of a 15 cm long *G. omarorum* was reconstructed with the aid of two pictures: one from above and the other from the side. The EO was simulated by 8 poles placed between the pectoral girdle and the tail. This is equivalent to having 7 dipoles whose time course was determined by measuring the electro motive force (EMF) of the Thevenin equivalent for seven contiguous segments of *G. omarorum* using the air gap method[22]. The positions of the 8 sources in the model correspond to those of the electrodes in the air gap method. Since at each instant total current is zero, the potential at infinite is null.

### Test of the model

In order to check the results of the model, we compare the voltage fields produced by the model with those obtained experimentally. For that, we immobilize the fish with a bridal veil in order to make the measurements. Experiments were performed following the guidelines of the CHEA (Comisión Honoraria de Experimentación Animal, ordinance 4332-99, Universidad de la República Oriental del Uruguay) and approved by the Animal Ethics Committee of the Instituto de Investigaciones Biologicas Clemente Estable (protocol number 001/03/2011).

We compared the model predictions with experimental measurements of the potential in the absence of objects. Results obtained with different water conductivities were also compared. Field potentials were measured between the intersection points of a rectangular lattice on the side of the fish and a fixed far reference. The recording points were scanned using an adapted plotter driven by a computer. Electrode tips were moved step-by-step along series of points on a previously defined plane using a computer controlled X-Y plotter (HP 7015A). This device allowed us a precise (less than 50 micrometers) control and recording of the active electrode position. Previously to the experiment, electrode trajectory was defined. On the side of the body equally spaced positions along a line following the skin were explored.

Signals were digitized at least at 20 kHz per channel and amplified enough to have at least 12 bits resolution (AM systems-1800, 10–10000 Hz band pass). Data acquisition was made in epochs of 550–700 ms, starting 100 ms after the electrode movement ceased. Five to seven channels were recorded in each experiment: a) the head to tail EOD recorded between two electrodes placed on the main axis of the fish at opposite faces of the tank; b) the local field (3 channels) and c) the potential), d) the X and Y positions of the electrode on the horizontal plane and e) a computer emitted trigger signal that started the acquisition after the electrode had reached each pre-programmed X-Y position. We used a head-tail recording to align temporally the potentials recorded at each point.

## Results

We will show first how the Model of *Gymnothus omarorum* (MoGO) reproduces experimental results. Afterwards, we show how the sources along the EO generate the field and how resistive objects distort this field generating images on MoGO's skin.

### Testing MoGO

For testing the model, we compare the simulations and experimental data obtained from the same scene, particularly: 1) the basal field and 2) the effect of water conductivity on the basal field.

**Figure 1:**
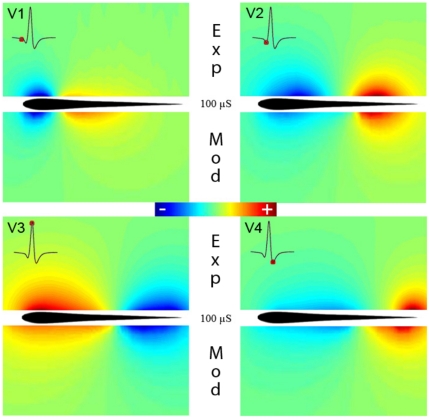
Comparison between experimental results and model predictions of the electric field generated by *G. omarorum*'s EOD for a 100 µS/cm medium conductance. The colormaps compare the fields of potential experimentally measured in a horizontal plane (upper half) at the peak of each component (V1 to V4, marked at the insets as a red dot in the head to tail EOD) with the same fields predicted by the model (lower half).

Test 1: Figure 1 compares the electric potentials produced by the model (lower pannels) with the experimentally determined (upper panels), for each component of the EOD (V1 to V4). The similitude between experimental and model results is evident. These results also show the changes in polarity which can be easily deduced by the head-tail measure (Fig. 1, insets). The result predicted by the model for the complete EOD can be found in Video S1.

**Figure 2:**
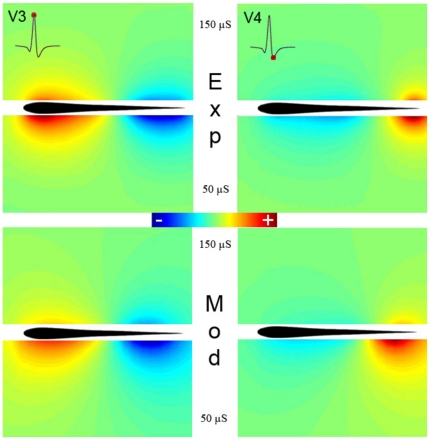
Comparison between the fields of the electric potential generated by *G. omarorum* and MoGO at two different conductances of 150 and 50 µS/cm. The upper pictures show the experimental results for V3 (left) and V4 (right) using two medium conductances, 150 µ/cm (upper halves) and 50 µS/cm (lower halves). The two lower pictures were the same results determined by the MoGO.

Test 2: we calculate the basal electric field with both, the model and the experimental setup for different water conductances. Figure 2 shows, that a decrease in medium conductance determines an increase of the electric field intensity. Small quantitative differences could be related to the electrocytes sensitivity to changes in the sources generated as a function of longitudinal currents [23]. In addition, for a lower conductance the field spreads further away. These results, shown only for V3 and V4, were verified for the complete discharge of *G. omarorum* (results not shown). This demonstrates that MoGO is suited for simulating complex scenes including changes in context.

### From sources to fields

In asynchronously firing distributed EO, the field in different points depends on both the time course of the source's amplitude and the position of the source. Obviously, since rostral sources are excited first and caudal ones later, the EO starts generating a larger field close to the head and afterwards the caudal sources will produce larger fields around the tail. But the size and shape of the field depends also on the position of a given source along the fish's body. For making this clearer, we studied how the fish shape and internal conductivity affects the generation of the basal field. The way in which sources inside the fish generate current densities on the fish skin is important, since these currents determine the field in the whole environment.

**Figure 3:**
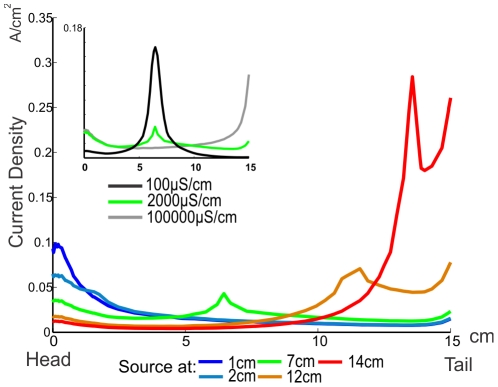
MoGO calculated transepithelial current density along a line on a horizontal plane. Different positions (color coded) of equivalent, unitary, sources are compared. The inset compares how spread of transcutaneous current density depends on body conductance. The source is placed at mid-body (7 cm from the head). Medium conductance was set at 100 µS/cm.

Figure 3 shows the profile of the current density (or its equivalent: the electric fields) generated by equal and individual sources placed in different positions on the midline of the fish's body. Sources located at different positions in the body of the fish generate different patterns of transepithelial density currents profiles along the fish skin. Note that the current densities tend to augment towards the tail and the head, following the decrease in surface area to both sides (edge effect). Our results show that sources placed near the head and in the abdominal area of the fish have an important effect on the generation of currents through the foveal region; hence, the contributions of abdominal sources to the EOD are important for electrolocation. Notice that, the contribution of the sources located between the tail and the middle of the fish, is significantly smaller than that of those near the head, yet since usually the amplitude of the sources increase caudally, rostral regions of the EOD (up, at least, to the middle of the fish) contribute similarly on the electric field at the fovea. In contrast, sources situated close to the tail contribute less to transepithelial currents through the fovea but may be important for electrocommunication. For example, a caudally located dipole doubles the weight of a rostrally located one of the same strength in the field measured between two points separated 10 cm respectively from the head and tail of a fish of 15 cm total length. This is an effect of both the shape and internal conductivity of the fish.

The inset in figure 3 shows the same currents produced by a source placed in the mid-body of fishes with high (black curve), normal (green) and low (gray) tissue conductivity. A high resistivity fish has a much localized effect on transepithelial currents. On the contrary, a highly conductive fish behaves like a metal object, with an homogeneous potential and where currents to the environment are ruled solely by the geometry of the fish with maxima at the head and tail (edge effect), and consequently larger than those near to the source. The similitude between transcutaneous current densities through the head, when the internal resistivity goes from normal to a low one, and the difference with high internal resistivity, shows that internal resistivity is close to an optimal for the generation of currents through the fovea by sources in the middle of the fish. This condition favors the generation of large currents through the fovea increasing its sensitivity.

### From instant fields to a spatiotemporal pattern

**Figure 4:**
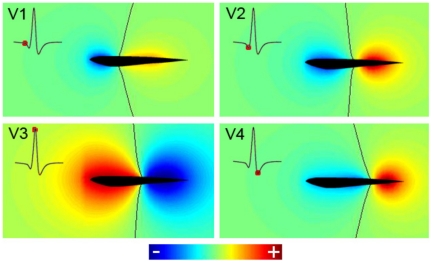
Normalized electric potentials, on the sagittal plane, at the peaks of the rostro-caudal potential difference. Each image corresponds to the instant when the head to tail EOD reaches the peaks of the waves V_1_, V_2_, V_3_ and V_4_. Black lines indicate the points where the potential is zero. Insets show the head to tail EOD, with red dots indicating the peaks of the 4 waves.

MoGO allows calculating the surrounding electric field for the 200 instants (steps of 0.04 ms) of a single EOD. To illustrate the results we plot the field potential, as a colormap, of a sagittal plane surrounding the fish: the gradient of this map is the electric field. To exemplify the data obtained for the whole EOD (see Video S1) we selected the peak times of the four main components of the discharge in the head to tail recording (V1, V2, V3 and V4; Fig. 4, insets). The black line indicates the points where the potentials are zero. Zero points form an unbounded surface (whose intersection with the sagittal plane is represented by the black lines in each panel). At some times in Video S1, a compact (bounded and closed) zero surface appears (See Text S1, in complementary information). When all the positive poles are placed rostrally (or caudally) to the negatives ones, all the zero points are on a single surface leaving positive and negative points in different sides. In other cases, another ovoidal zero surfaces may appear. In our simulations these are not shown (for most frames), because they present themselves inside or very close to the fish. However, in some frames this ovoidal “zero” surface is large enough to be detected (see Video S1 and Text S1). In most frames, the external potential is similar to that of a dipole, where the black line shows points where the potential is zero and the field is almost parallel to the longitudinal axis of the fish. Note that this zero line moves backwards during the EOD. The result is similar for all the planes through the longitudinal axis of the fish (results not shown).

**Figure 5:**
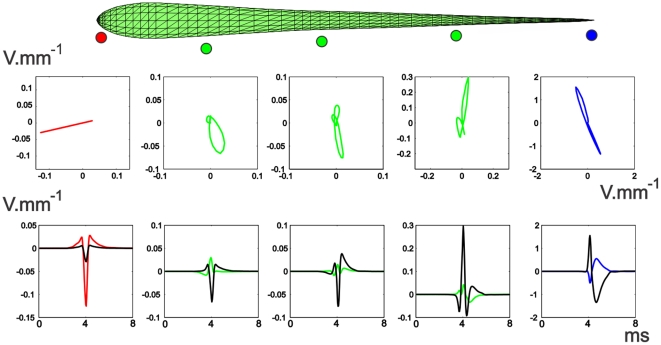
Pericorporal Local Electric Field. **Top:** The time course of the electric field along the EOD, at different points on a horizontal plane, near the lateral skin of the fish. The colored dots indicate schematically the position in space where the field was calculated. **Bottom:** LEOD components at the same positions of the electric field. Colored traces correspond to longitudinal values and black traces to transversal values.

**Figure 6:**
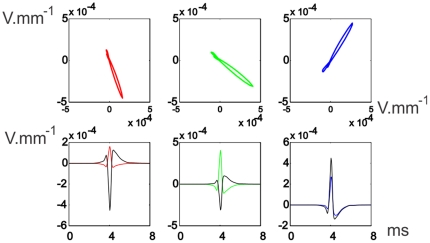
Far-off Electric Field. **Top** Field time course for 3 points at 1 fish distance from the lateral skin of the fish and with the same projections along the longitudinal axis of the red, rostral green and blue points of Figure 5. **Bottom** LEOD values for these three points: colored traces correspond to longitudinal values, while black traces correspond to transversal values. All calculated in a horizontal plane.

These spatial changes of the field potential along the EOD affect differently the waveform of LEOD around the fish. Figure 5 shows that the pericorporal electric field close to the head is oriented in an almost fixed direction, but the direction changes close to the trunk and tail. Far from the fish, the field in each point is oriented also in a fixed direction as that produced by a dipole (Fig. 6). These results were expected since: 1) In rostral regions (very close to the skin), the field should be almost perpendicular to the skin due to the large conductivity of the fish body in relation to that of the surrounding water [16], and consequently the direction of the field is almost fixed. 2) Close to the regions where the zero potential surface appear and where the perpendicular direction is far from uniform, the field changes in direction. 3) Far from the fish (Fig. 6) the position of the dipoles (in relation to the recording point) is almost the same and, consequently, the potential can be calculated considering the dipoles are in the same point: this approach is widely used in the interpretation of the Electrocardiogram [24].

At the head region and far from the fish, the field is collimated and coherent as in those modeled for *G. petersii*, generating electric images of objects and communication signals in a similar way. At the trunk and tail region this similarity does not exist, green and blue traces, in figure 5, show how the direction of the field changes throughout the EOD.

### From Basal fields to Images

MoGO allows us to examine the mechanism of image formation in *G. omarorum*. With that objective in mind we studied the characteristics of the image as a function of the position of an object.

**Figure 7:**
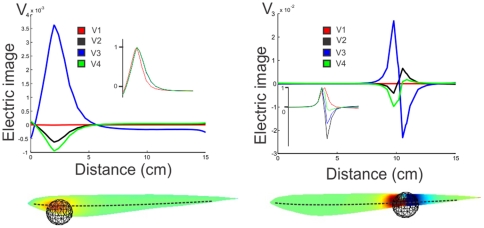
Images of a metal sphere. **Left**: when the sphere is in the head region; **Right**: when it is close to the place where the field at the peak of V_3_ is zero. **Top:** images calculated along a line through the horizontal plane by the center of the sphere. Insets show the normalized images. **Bottom:** images on the skin of the fish, indicating the position of the sphere is shown by its image. The broken line on the fish indicates the line along which top images were plotted.

Figure 7 shows the image of a metal (highly conductive) sphere on the skin, calculated with the MoGO. The electric image was calculated as the difference between the transcutaneous currents with and without the sphere. As expected by the changes in the electric field already described, the image of an object will be a function of both, the point on the skin and time.

We calculated these images for a metal sphere when placed at both, a rostral and a caudal region. Figure 7 presents, at left bottom, a scene including a sphere situated near the head of the modeled fish, with the image at the time corresponding to the peak of V3, represented as a colormap on the skin of the fish. At left top, we compare the longitudinal profiles along the dotted line of the corresponding electric image to the four principal components of the EOD (V1 through V4). It shows the proportionality between these Mexican hat profiles (inset); actually, this proportionality holds for all the images on the skin at different times along the EOD.

**Figure 8:**
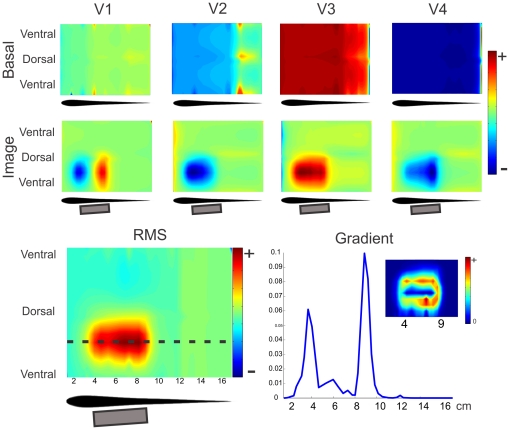
Image of a large metal object. Two dimensional plots correspond to the images on the skin, where it was cut through the ventral intersection with the saggital plane, stretched perpendicularly to the longitudinal axis, to cover a rectangle. The horizontal scale corresponds to the distance in cm from the frontal tip along the skin through the horizontal plane. **Top:** Images generated on the skin of MoGO by a 2 by 5 cm cylinder placed almost parallel to the lateral skin at different instances of the EOD corresponding to the peaks of the four main components. Top Row: images, at the head and trunk regions, of the basal currents for each wave. Bottom Row: the electric image (at the same region) as the difference between currents with and without the cylinder. **Bottom Left:** RMS image of MoGO's body. **Bottom Right:** Longitudinal profile of the gradient of the RMS electric image showing two peaks signaling the position of the edges of the object. **Inset:** 2D mapping of the gradient of the RMS image shows the correlation between high gradient and the edges of the object.

We compared these results to a simulation in which the sphere is moved closer to the tail (Fig. 7 right). At top, the images along the dotted line show that in this case the images generated at V1 through V4 are not proportional. In this case, for V2, V3 and V4, the images are biphasic, although not proportional. As we have shown previously, the discharge of *G. omarorum* is similar to that produced by a unique dipole that changes position and amplitude through time. This change in the position of the dipole during the EOD could be causing this distortion of the image. When the moving dipole passes by the spot where the sphere is located, it is placed in a variable and far from uniform field and, consequently, the image on the skin changes in a non proportional way. So, in a sense, as the dipole runs by the position of the sphere, the image changes drastically. This change in the image formation for the abdominal skin surface is not detrimental for perception. The four main components of the EOD “illuminate” differently an object, generating distinct electric images on the skin of the fish (Fig. 8 Top). In this sense the different components add to a general perception of the object (Fig. 8 Bottom Left).

### Images of large objects

A cylinder (2 cm of diameter and 5cm long), on the side of the fish at the trunk and tail region, generates images that differ for the waves of the EOD (Fig. 8). By comparing the image with the basal colormaps one can evaluate the changes in each part of the wave (Fig. 8 top rows). V1 shows a rostral-caudal biphasic pattern meaning an increase of V1 at the rostral and a decrease at the caudal portions of the object. V2 shows an increase all along the projection of the object with a maximum in front of the rostral region and vanishing at the caudal end of the cylinder. V3 increases occur in front of the whole cylinder with a rostral maximum. V4 shows a rostro-caudal increase on the central projection of the object. The dorso-caudal profile also changes along the cylinder projection increasing in extension from rostral to caudal. Note that in all the cases, for the central zone of the image the color (red or blue) is the same that the color of the basal field in that zone, indicating an increase in the absolute value of the basal field. In V1, since the basal field generates negative currents at rostral regions (cold tones), the blue colors of the image indicates an increase of the negative basal currents; by the contrary the red colors in the image correspond to positive basal currents (warm tones), it indicates an increase of the positive basal currents: in every place the absolute value of the current increases.

The temporal variations of the spatial pattern of the image induce us to find a unique temporal image representing the variations of the LEOD at the different points on the fish skin. With this objective, we evaluate the pattern of the change in energy as the RMS of the image in time (Fig. 8, bottom-left colormap): for each point on MoGO's surface we calculate the RMS image as the squared root of the average of the squared current differences for all instants along the EOD. It clearly portrays the object's foot print facing the skin. The gradient of this image shows that limits of the cylinder as it is illustrated in the rostrocaudal gradient profile that clearly delimits the rostral and caudal edges of the cylinder (Fig. 8, bottom-right colormap and plot).

Large images of nearby objects are placed (at least partially) outside the head region and, consequently, they are illuminated from different angles, similarly to what happens in the trunk-tail region. For this reason, the examination of a large object by electric fish will be made mainly by the trunk to tail region. Nevertheless, local characteristics as edges may be examined in detail by the fovea.

## Discussion

We introduce a 3D realistic model of electrolocation of *G. omarorum*, taking into account fish geometry and distributed electric sources. These aspects are important in different ways. Firstly, since longitudinal internal resistance varies inversely with the square of the diameter and skin conductance varies proportionally with the diameter, the sources placed in different regions of the fish have different effects on object “illumination” and also different weight on the basal field. As figure 3 shows, the potential produced by sources of the same intensity located at different regions of the fish spreads differently. Secondly, different sources contribute with different waveforms and amplitudes. Then, MoGO allows us to adequately portray the effect of fish geometry and the time course of the sources in field and image generation.

### Testing of MoGO

The MoGO allows the calculation of the electric potential fields generated at any moment during the EOD (Fig. 4 and Video S1), showing how they change in amplitude and shape. For the peaks of the different components of the head-tail EOD, the maps of electric potential are different in magnitude and location of the reversal point. This results from the different weights of the active components at each of the head to tail peaks. This different weight results from two factors: the relative magnitudes of the local electromotive forces and the relationship between the water and tissue resistance at different regions of the body.

### How does a complex EO determine image formation?

*G. omarorum*'*s* EO is complex (in relation to other weakly electric fish) in several aspects. This is caused by the fish's geometry and the relative magnitude of distributed sources that contribute differently in the polarization field at different regions. The density and size of the electrocytes decrease exponentially from head to tail and the diameter decreases in the same direction. Therefore, the equivalent source of the abdominal region is characterized by low electromotive force and low internal resistance while the equivalent source of the tail region is characterized by high electromotive force and high internal resistance. The central region is intermediate. Thus, the field generated at the vicinity of the head, is dominated by the abdominal region and the far field is dominated by the central and tail regions. This is because the field generated at the abdominal region extends less than the field generated at the rest of the body. Differential attenuation and the asynchrony of the excitation of different regions of the EO leads to a time course of the field that varies along the fish (as shown in Fig. 5).

In general, an electric image of a **resistive object** is a function of both time and place on the skin. In *G petersii* [21], it is defined by a function with a constant spatial shape on the skin and that at each point during the EOD's time course is proportional to the amplitude of the head to tail EOD. In other words, the image is given by the spatial shape of the image multiplied by the time course of the EOD amplitude (temporal shape). Since the temporal course is the same for every point on the skin, the image is two dimensional: the stimulus on each point of the skin may be defined by the LEOD at the maximum of the EOD. This is due to the fact that in *G petersii* the EO is concentrated at the tail and, therefore, it acts as a single source generating a coherent field everywhere. By the contrary, in *G omarorum* the image should be described as a function of three independent variables: time and two coordinates defining the position on the skin. Actually, it is a three dimensional function.

In vision a fixed image may be considered as an infinite dimensional function of the place in the retina and the wavelength spectrum (two variables for the place in the retina and infinite for the wavelength). But, in the case of an animal without color vision (with only one type of photo-receptors), the intensity of the image in each point is given by a single number as in a black and white picture: a map of light spectra on real numbers (the transformation of the incident energy in receptor potential). Then, the image may be considered a two dimensional map on the retina.

Electric images of **capacitive objects** are more complex. Since electric fish eat alive prey which present capacitive properties [25], it is important to characterize the images of capacitive objects. Hence, the field polarizing the prey around the electric fovea (placed on the head, [26]) should be suitable for impedance analysis.

Capacitive objects behave as filters, generating a perturbing potential that is not proportional to the basal potential, but depends on its preceding time course. This is because the charges of the capacitances determine partially the perturbing potential. These charges depend on the direction of the basal field and if this direction is variable, charge distribution will change in time, producing perturbing fields changing in both, amplitude and shape. Consequently, the images will move on the fish skin, making their sensory processing a difficult task for the fish nervous system. Then a basal field, suitable for the perception of capacitive objects would be coherent and collimated.

### From Fields to Images

The characteristics of the field condition those of the image. MoGO allows us to define at least 3 regions of the surrounding media of the fish with important differences in the characteristics of the basal field and, consequently, in the way images are generated.

**Near to the fish at the head**, the field is coherent and collimated, as happens everywhere in *G petersii,* a fish with a concentrated EO at the tail. Consequently, the way images of objects in this region are generated follows similar rules. A small resistive object, polarized by the electric field, generates a perturbing field and an image with a fixed shape and with the same time course of the EOD.

Since the LEOD in the frontal regions have similar temporal shapes and consequently, at the fovea and adjacent regions, resistive objects mainly cause changes in amplitude, without large variations of time course. Nevertheless, the picture is different for large enough objects when the field around it is not coherent. In this case, the LEOD changes its time course, even for resistive objects.

Capacitive objects change the time course of the LEOD, stimulating different electroreceptors in the skin differentially, making possible the sensing of a qualia in *G petersii*, named by our group as electric color [21].

In the head region of *G omarorum*, capacitive objects produce clear changes in the time course of the transepidermal voltage that can be detected differentially by the three types of receptors, making possible the discrimination of electric color [27].

Then, since the images of objects are produced in areas of the skin with more complex types and more density of electroreceptors this region behaves similarly to that of *G petersii*, making the task of detection of electric color relatively simple. This similitude between two types of electric fish indicates that they evolved convergently not only producing a similar sensory system, but also developing similar characteristics to produce images. It is sensible to think that this similitude is not constrained to the way images are produced but also to the processes taking place in the nervous system. Since the organization of the electroreception pathway is quite different in gymnotids and mormyrids, this processing may yield similar results using non identical mechanisms. Electric color may be coded by computing the modification of the time course of the local EOD, produced by capacitive objects [20], [17]. As in vision, the electric fovea and adjacent regions of *G omarorum* have at least three types of receptors. Since each receptor type has a different tuning curve and phase sensitivity [28], the qualia (*i.e.*: electric color) potentially perceived by this specie is richer than in *Gnathonemus petersii* [22]. But, as we have described, large objects may produce changes in the LEOD time course. This may cause confusion in the perception of electric color.

Since the head constitutes an edge of the fish body, the EOD generates there a large local field facilitating the examination of very close objects. Because of the edge, the neighboring field is highly divergent. Consequently, it decays rapidly and the sensory system looses discrimination with distance generating “myopia”. Far away only large objects can be detected by electroreceptors on the head.

We conclude that the head region is specially suited for the examination of small close objects, particularly those eatable and alive. The possibility to process color and details of small, very close objects is thanks to the high density and variety of electroreceptors in this region. This region may also participate in the location of large objects orienting the fish navigation to approximate them and to further examine them.

### Near to the fish at the trunk

In points of this region the field components do not vary proportionally, since the direction of the field changes along the EOD. This allows the “illumination” of large objects from different directions. Consequently, objects in these regions will produce spatial profiles changing in shape and amplitude along the EOD.

But in this region almost all the electroreceptors are of the same type and, consequently they respond to a unique parameter of the stimulus. In the absence of experimental results about the sensibility of these receptors we propose the total energy during the EOD (the RMS) as a candidate for this parameter. Of course other parameters, as the peak to peak amplitude, are equally likely to be the most adequate. The resulting image is a more faithful representation than the one achieved by illuminating the object from a local source, a procedure valid in *G. petersii* (Fig. 8).

Considering the whole EOD integrated in time (RMS values) the resulting image is a more faithful representation than the one achieved by illuminating the object from a local source as in *G. petersii* (Fig. 8). When fish explore objects by smooth swimming using the anal fin the gradient of such image may be computed by the central nervous system to define its limits.

This advantage takes a toll on the capability for complex impedance discrimination. Since the effect of capacitive objects is a distortion of the LEOD time course, in this region, it will be a difficult task for the nervous system to detect the presence of capacitive objects. Besides, images of objects in this region project on skin covered by a single type of sparsely distributed receptors. This implies that, at caudal regions, capacitance encoding would be either not possible or has to be encoded by different characteristics of the electroreceptors response.

We conclude that this region is specially suited to determine the shape or other properties of large objects. Probably it can't determine qualia of objects as color or texture, but general shape, edges, etc.

**Far from the fish**, the field is quite collimated (as produced by a single dipole with variable amplitude). Since the field is quite small, the perturbing field will also be small and since it decays with the distance to the object, in the fish skin its value will be even smaller. Consequently objects comparable in size to the fish placed in this region would produce electric images too small to be perceptible: this region is outside of the active electric sense discrimination bubble.

Thus, these far fields could be used for two purposes for finding large contextual elements as for example a wall of the tank or for electro-communication. They also can be used by fish to track a conspecific. In far field region the direction of the field is collimated and therefore following the direction of the field can lead unequivocally to the position of other fish [29]).

### Conclusion

In this paper, we introduce a model for the computation of electric fields and images in a weakly electric fish with a distributed electric organ: *Gymnotus omarorum*. Model results were checked against experimental ones, resulting in a strong qualitative similarity. In addition, we obtained general results about fields and images, producing a general picture of them in fishes with distributed organs, stressing the importance of the rostro-caudal sequence of the excitation of the electric organ and the shape of the fish body. We show that in these types of fish, the image is a spatio-temporal pattern.

Near fields define an active electrolocation bubble that moves together with the fish for exploring the environment. Far fields outside such bubble cannot be used for active electrolocation but may serve for tracking or communication purposes.

Around the head, where the fovea is situated the basal field only varies in amplitude making this region suitable for examination of small objects and color perception. This characteristic is shared by an electric fish with a concentrated electric organ as *G petersii*, indicating that the mechanisms of the detection of distortions of the LEOD produced by capacitive objects (i.e.: the perception of electric color) may be shared for both species. These characteristics induced different researchers in the field to compare this region with the fovea of the eye [30], [31]. Nevertheless, the fovea in vision receives a neat image of the object, but in electrolocation, the image is neat only for very close small objects. Probably, this sensory surface may be better compared with the fingertips in touch. In both cases there are neat images of very close small objects, they are sensitive to qualia (electric color in our case and texture in touch), and requires the motor system to provide the energy carrier (limb movement for touch, and electrogeneration in our case).

The field around the trunk is neither collimated nor coherent but objects are more uniformly illuminated considering the whole energy content of the EOD. This makes this region suitable for object representation allowing the fish to explore their limits using longitudinal movements. If we compare the head with the fingertips we should compare the trunk with the hand palm: both can adapt its shape to be close to the object along a large portion and generate an image with weak discrimination. To determine the characteristic of the object the fish should integrate electrolocation with propioception as in haptic systems. Within the framework of comparative cognitive sciences, we propose that active electrolocation contributes to haptic perception as active touch does in other species.

## Supporting Information

Video S1 **Complete electric organ discharge.** Electric field potential generated by the electric organ discharge represented on a sagittal across the modeled fish. Hot tones represent positive potential values, while cold tones represent negative electric potentials. The black line indicates the points where the potentials are zero. The video shows several times the electric field generated by the MoGO during the timecourse of the EOD.
(M1V)

Text S1 **Why fish with distributed organs can have more than one reversal (“zero crossing”) surface.** This text briefly explains the existence of several reversal surfaces for the electric field generated by MoGO.
(DOCX)
