# Supplementary material for: Fish Geometry and Electric Organ Discharge Determine Functional Organization of the Electrosensory Epithelium
Source: PLoS One. 2011 Nov 11;6(11):e27470. doi: 10.1371/journal.pone.0027470 (PMC3214058; doi:10.1371/journal.pone.0027470)
Supplement: Text S1 — Why fish with distributed organs can have more than one reversal (“zero crossing”) surface. This text briefly explains the existence of several reversal surfaces for the electric field generated by MoGO. (DOCX) [file pone.0027470.s002.docx]

**Why fish with distributed organs can have more than one reversal (“zero crossing”) surface**

The EO in *G omarorum* is a ventral series of electrocytes, whose rostral and caudal membrane activation produces a series of dipoles during each EOD[1]. Then, we represented the EO as a distribution of either dipoles or sources, along a rostro-caudal line: actually, the distribution of sources is the derivative of the distribution of dipoles. Let us assume, for example, that the line is a straight line and that the distribution of dipoles is uniform and are pointing in the same direction: then, the poles consist of two poles at each extreme: the potential will be zero at the middle point. If the sources distribute on the line as two separate groups of different sign, there is one zero potential surface separating sources of different sign. If the sources distribute on the line as three separate groups with alternate signs, there are two zero potential surfaces to separate sources of different sign: in general, one of the surfaces is unbounded and the other will be compact (closed and bounded, as a spherical surface). In very particular cases (that are not expected to be found in nature), both surfaces are unbounded, as for example, when there are 3 sources aligned and equally spaced with currents -1, 2 and -1. But, in general, this situation does not happen. The topology of these surfaces is extremely sensible to the values of the parameters: if they are slightly changed the solutions may change radically; it is not structurally stable. In the general case, both surfaces are closed but one of them is unbounded and the other is bounded: one is an infinite surface (as a plane) and the other is similar to an ovoid. If there are *n* transitions of the sign of the sources, there may be up to *n* zero surfaces that do not intersect each other, and where, in general, only one is unbounded.

1. Lorenzo D, Velluti JC, Macadar O (1988) Electrophysiological properties of abdominal electrocytes in the weakly electric fish Gymnotus carapo. J Comp Physiol 162: 141-144.
